# Biomechanical diagnostics of the cornea

**DOI:** 10.1186/s40662-020-0174-x

**Published:** 2020-02-05

**Authors:** Louise Pellegrino Gomes Esporcatte, Marcella Q. Salomão, Bernardo T. Lopes, Paolo Vinciguerra, Riccardo Vinciguerra, Cynthia Roberts, Ahmed Elsheikh, Daniel G. Dawson, Renato Ambrósio

**Affiliations:** 1 Rio de Janeiro Corneal Tomography and Biomechanics Study Group, Rio de Janeiro, Brazil; 2 Instituto de Olhos Renato Ambrósio, Rua Conde de Bonfim 211 / 712, Rio de Janeiro, RJ 20520-050 Brazil; 3 Department of Ophthalmology, Hospital São Vicente de Paulo, Rio de Janeiro, Brazil; 4 Brazilian Study Group of Artificial Intelligence and Corneal Analysis – BrAIN, Rio de Janeiro & Maceió, Brazil; 5 Department of Ophthalmology, Federal University of São Paulo, São Paulo, Brazil; 6 Instituto Benjamin Constant, Rio de Janeiro, Brazil; 7 School of Engineering, University of Liverpool, Liverpool, L69 3GH UK; 8 Department of Biomedical Science, Humanitas University, Rozzano, Italy; 9 Eye Center, Humanitas Clinical and Research Center, Rozzano, Italy; 10 Department of Ophthalmology, Humanitas San Pio X Hospital, Milan, Italy; 11 Department of Ophthalmology and Visual Science, Department of Biomedical Engineering, The Ohio State University, Columbus, OH USA; 12 School of Biological Science and Biomedical Engineering, Beihang University, Beijing, China; 13 NIHR Biomedical Research Centre for Ophthalmology, Moorfields Eye Hospital NHS Foundation Trust and UCL Institute of Ophthalmology, London, UK; 14 The University of Florida Department of Ophthalmology, Gainesville, FL USA; 15 Department of Ophthalmology, Federal University the State of Rio de Janeiro (UNIRIO), Rio de Janeiro, Brazil

**Keywords:** Corneal biomechanics, Corneal ectasia, Corneal imaging

## Abstract

Corneal biomechanics has been a hot topic for research in contemporary ophthalmology due to its prospective applications in diagnosis, management, and treatment of several clinical conditions, including glaucoma, elective keratorefractive surgery, and different corneal diseases. The clinical biomechanical investigation has become of great importance in the setting of refractive surgery to identify patients at higher risk of developing iatrogenic ectasia after laser vision correction. This review discusses the latest developments in the detection of corneal ectatic diseases. These developments should be considered in conjunction with multimodal corneal and refractive imaging, including Placido-disk based corneal topography, Scheimpflug corneal tomography, anterior segment tomography, spectral-domain optical coherence tomography (SD-OCT), very-high-frequency ultrasound (VHF-US), ocular biometry, and ocular wavefront measurements. The ocular response analyzer (ORA) and the Corvis ST are non-contact tonometry systems that provide a clinical corneal biomechanical assessment. More recently, Brillouin optical microscopy has been demonstrated to provide in vivo biomechanical measurements. The integration of tomographic and biomechanical data into artificial intelligence techniques has demonstrated the ability to increase the accuracy to detect ectatic disease and characterize the inherent susceptibility for biomechanical failure and ectasia progression, which is a severe complication after laser vision correction.

## Background

### The cornea and its biomechanical behavior

Along with the tear film, the cornea is the first optical interface of the visual system and is responsible for most of the refractive convergence power of the eye. This transparent avascular tissue also acts as a barrier against trauma and microbial agents [1, 2]. Typically, the cornea is thinner at the center and presents a gradual increase towards the periphery. Previous studies have demonstrated a normal distribution in healthy eyes, with an average central corneal thickness of 545 μm (standard deviation of 35 μm; range, 440–650 μm) [2, 3].

Remarkably, the cornea presents a delicate and complex balance between stiffness, strength, extensibility, and overall toughness to bear and endure the internal and external forces that continuously stress it, distort its shape, or threaten its integrity. Laboratory studies found higher corneal stiffness following the direction of the collagen fibrils (longitudinal x- and y-axis) than perpendicular to them (shear, radial, or z-axis) [4]. While the contributions of the epithelium, Descemet’s membrane, and endothelium are relatively weak, and the contribution from Bowman’s layer is still controversial, the stroma is responsible for most of the corneal strength [4]. Furthermore, the anterior 40% of the corneal stroma is the strongest region, whereas the posterior 60% of the stroma is at least 50% weaker according to tensile strength studies in human donor corneas [2].

The cornea also has viscoelastic properties that allow for its functioning as a biological mechanotransducer of stress. Viscoelastic behavior is complex as it means the tissue response is dependent on the strain rate, which influences the deformation in the cycle of loading/unloading. The system experiences a gradual increase in strain under sustained load so that the energy dissipation is related to the viscous sliding of the fibrils and lamellae in a hydrated proteoglycan matrix [1].

## Main text

### Clinical applications of corneal biomechanics

Corneal biomechanics emerged as a relevant topic for research and development in modern ophthalmology because of the many potential applications [5]. In the glaucoma field, the relevance of biomechanical properties for intraocular pressure (IOP) measurements was extensively investigated [6–8]. Moreover, since the Ocular Hypertension Treatment Study (OHTS), corneal parameters including (and beyond) central corneal thickness represent significant predictors for the development and the severity of glaucomatous optic neuropathy. Corneal biomechanics might further be a significant confounding factor for IOP measurement that should be considered in clinical decision-making [9–11].

On the subject of ectatic corneal diseases, such as keratoconus (KC), and pellucid marginal degeneration, knowledge of corneal biomechanics offers a significant contribution and relevance for the diagnosis, staging, and prognosis of the disease [12–14]. Understanding the cornea’s biomechanical behavior is relevant for the detection of subclinical KC as well as for detection of ectasia progression, while changes in topography are still insufficient to provide conclusive evidence [15]. Additionally, the biomechanical investigation has become significant in the setting of refractive surgery to identify patients at higher risk of developing iatrogenic ectasia after laser vision correction, along with enhancing the predictability and efficacy of these elective procedures [11, 15–17].

This review discusses the latest developments of corneal biomechanics investigation, particularly in the detection of mild ectatic disease.

### Evolution of corneal imaging and characterization

Corneal shape imaging technologies have been improperly considered surrogate methods for the evaluation of corneal biomechanical properties [18]. Nevertheless, while this is possible to assume that corneal shape reflects biomechanical properties, for the proper assessment of biomechanical response, an applied load is needed.

Biomechanics cannot be assessed from a single image without a perturbation of some kind. The concept of multimodal corneal imaging was introduced to pivot the many diagnostic tools available [14]. Placido disk-based corneal topography has been proven to improve the ability to detect abnormalities of mild corneal ectasia in patients with normal distance-corrected visual acuity and unremarkable slit lamp examinations [13, 19, 20]. Subsequently, the advent of anterior segment tomography, with the 3-dimensional reconstruction of the cornea, provided more detail about corneal architecture with a variety of quantitative indices derived from the front and back elevation and the pachymetric maps [3, 14, 21, 22]. The ability of corneal tomography to further enhance the accuracy of detecting mild or subclinical ectatic disease was demonstrated in different studies involving eyes with typically normal topography from patients with clinical ectasia identified in the fellow eye [13, 23–28]. Such cases with regular topography from patients with very asymmetric ectasia (VAE-NT) represent the most important model for developing and testing novel diagnostic strategies for enhancing ectasia detection [14]. Moreover, corneal tomographic parameters revealed a superior ability to recognize susceptibility to develop ectasia after LASIK in retrospective studies involving patients with such a complication [21, 29, 30].

Segmental tomography with epithelial thickness was established initially with very-high-frequency ultrasound (VHF-US) [31–34], but was later made conceivable and popularized by spectral domain optical coherence tomography (SD-OCT) [26, 35–37]. However, the need to go beyond corneal shape evaluation for depicting ectasia risk within the biomechanical domain has been supported and promoted [38, 39].

### Ocular response analyzer

**Fig. 1:**
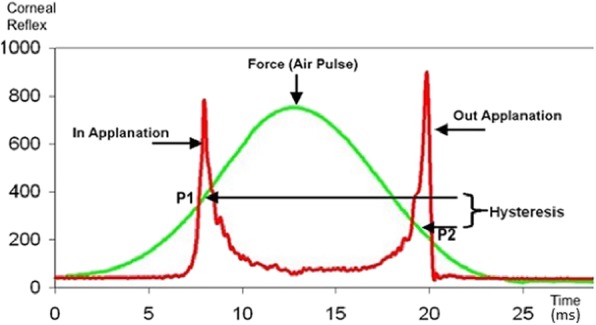
Ocular response analyzer (ORA) measurements showing the air pulse deforming the cornea (ingoing phase) and registering corneal signal (Y axis) through time (X axis) in milliseconds, in which P1 is the first applanation moment. The Gaussian configuration is from when the air pulse signal is shut off, then with the continuing increase in magnitude of the air pulse due to inertia in the piston, the cornea assumes a concave configuration. In the outgoing phase (air pressure decreases), the cornea passes through a second applanation, when the pressure of the air pulse (P2) is again registered. The pressure-derived parameters generated are corneal hysteresis (CH) and corneal resistance factor (CRF). This figure is a composite made by the authors of classic pictures available in public domain

In vivo measurements of corneal biomechanical response first became available with the introduction of the ORA (Ocular Response Analyzer; Reichert Ophthalmic Instruments, Buffalo, NY) in 2005 [39, 40]. The ORA is a non-contact tonometer (NCT) with a collimated air puff to indent a central 3–6 mm apical corneal area. An advanced electro-optical system monitors the bi-directional movement of the cornea through its reflection of an infrared beam [40–42]. As the air pulse is activated, the cornea deforms in an inward direction (ingoing phase), passing through a first applanation moment, when the pressure (P1) is registered. At first applanation, the air pump receives a signal to shut off, the inertia in the piston allows the pressure to continue to increase so that the air pulse has a Gaussian configuration. The peak of the air pressure pulse is strongly influenced by P1, making it a key parameter for each ORA measurement. As the air pressure continues to increase, the cornea assumes a concave configuration. The outgoing phase starts as the air pressure decreases, allowing the cornea to return to its original shape gradually. During the outgoing phase, the cornea passes through a second applanation, when the pressure of the air pulse (P2) is again registered. The pressure-derived parameters *generated* by the standard ORA software are corneal hysteresis (CH) and corneal resistance factor (CRF; Fig. 1). CH is the difference between the P1 and P2 values, whereas CRF is calculated according to the formula: *a* [P1–0.7P2] + *d*, where *a* and *d* are calibration and regression constants to maximize correlation with central corneal thickness [40, 43].

Even though studies have reported CH and CRF to be lower in KC compared to healthy corneas [44], a considerable overlap in the distributions of both parameters was observed so that the sensitivity and specificity for KC diagnosis are relatively weak (Table 1) [45–47]. Further research found more accurate ectasia detection when analyzing the ORA waveform signal and developing new parameters that are related to the deformation response of the cornea during the NCT measurement [45, 48–50]. More recently, the integration of these new parameters with tomographical data demonstrated improved accuracy to detect mild or early ectatic disease [27]. Also, the waveform-derived parameters were found to document corneal biomechanical changes after crosslinking procedures in KC, while CH and CRF did not detect significant differences [27, 51].

**Table 1:** Ocular response analyzer (ORA) clinical study [45]

Parameter	NE (n)	Clin Ectasia (n)	Cut-off	Sensitivity (%)	Specificity (%)	AUC	95% CI
p2area	112	41	≤1554.438	80.5	96.4	0.939	0.888 to 0.971
p1area	112	41	≤2865.500	82.9	89.3	0.929	0.877 to 0.965
CRF	112	41	≤8.600	87.8	80.4	0.895	0.835 to 0.939
CH	112	41	≤8.700	75.6	86.6	0.852	0.786 to 0.904

*NE=*  normal eyes, *AUC=*  area under the receiver operating characteristics curve, *CI=*  confidence interval, *p1area* = area under the waveform peak during the first applanation, *p2area=*  the area under the waveform peak during the second applanation, *CH=*  corneal hysteresis, *CRF=*  corneal resistance factor

### The Corvis ST

The Corvis ST (Oculus, Wetzlar, Germany) is also a NCT approved by the United States F.D.A. (Food & Drug Administration) for tonometry and pachymetry. Internationally, this is also approved as a toll for biomechanical assessment of the cornea. During its measurement for biomechanical assessment of the cornea, similar to what happens in the ORA exam, the cornea deforms inward and outward while passing through two applanation moments. However, the Corvis ST has two fundamental differences from the ORA. First, instead of using the reflection of the infrared beam to monitor the deformation of the cornea, it uses an ultra-high-speed Scheimpflug camera that takes 140 horizontal 8 mm frames over a period of 33 ms. This approach allows a more detailed evaluation of the deformation process. Also, unlike the ORA, the Corvis ST yields a fixed maximal peak pressure for the air puff in every examination [52].

The Corvis ST calculates corneal deformation parameters based on the dynamic inspection of the corneal response (Table 2). By way of air pressure, the cornea begins to deflect in the backward direction. Whole eye motion is instantaneously initiated with a slow linear increase also in the same backward direction and then increases dramatically when the cornea reaches maximum displacement. Dynamic corneal response (DCR) parameters thereby either include or compensate for the whole eye motion. The parameters described as “deformation” are those in which whole eye motion is not compensated, while the “deflection” parameters take into account and compensate for the displacement of the eye. The deformation amplitude (DA) refers to the displacement of the corneal apex in the anterior-posterior direction and is determined as the most considerable dislocation of the apex at the highest concavity (HC) moment. The DA Ratio 1 or 2 mm is the central deformation divided by an average of the deformation 1 to 2 mm at either side of center with maximum value, just prior to the first applanation. Applanation lengths (AL) and corneal velocities (CVel) are recorded during ingoing and outgoing phases. The radius of curvature at the highest concavity (curvature radius HC) is also documented, and the integrated inverse radius is reciprocal of the radius during the concave state of the cornea. One should note that a greater concave radius is associated with greater resistance to deform or a stiffer cornea. Therefore, the higher the integrated inverse radius and maximum inverse radius, the less resistance to deformation and lower corneal stiffness. Corneal thickness, the standard Goldmann-correlated IOP, and a biomechanically compensated IOP are registered as well [53, 54].

**Table 2:** Corneal deformation parameters provided by the Corvis ST

Corvis ST – Parameters	
1st Applanation	The first applanation of the cornea during the air puff (in milliseconds). The length of the applanation at this moment appears in parenthesis (in millimeters).
Highest Concavity	The instant that the cornea assumes its maximum concavity during the air puff (in milliseconds). The length of the distance between the two peaks of the cornea at this moment appears in parenthesis (in millimeters).
2nd Applanation	The second applanation of the cornea during the air puff (in milliseconds). The length of the applanation at this moment appears in parenthesis (in millimeters).
Maximum Deformation	The amount (in millimeters) of the maximum cornea deformation during the air puff.
Wing Distance	The length of the distance between the two peaks of the cornea at this instant (in millimeters).
Maximum Velocity (in)	Maximum velocity during the ingoing phase (in meters per seconds [m/s]).
Maximum Velocity (out)	The maximum velocity during the outgoing phase (in meters per seconds [m/s]) .
Curvature Radius Normal	The cornea in its natural state radius of curvature (in millimeters).
Curvature Radius HC	The cornea radius of curvature at the time of maximum concavity during the air puff (in millimeters).
Cornea Thickness	Measurement of the corneal thickness (in millimeters).
Integrated Inverse Radius	Inverse of the radius of curvature during concave phase of the deformation.
Deformation Amplitude Ratio 1 or 2 mm	The central deformation divided by an average of the deformation 1 or 2 mm at either side of center with maximum value just prior to 1st applanation.
IOP	Measurement of the intraocular pressure (in millimeters of Mercury [mmHg]).
bIOP	Biomechanically-corrected IOP

**Fig. 2:**
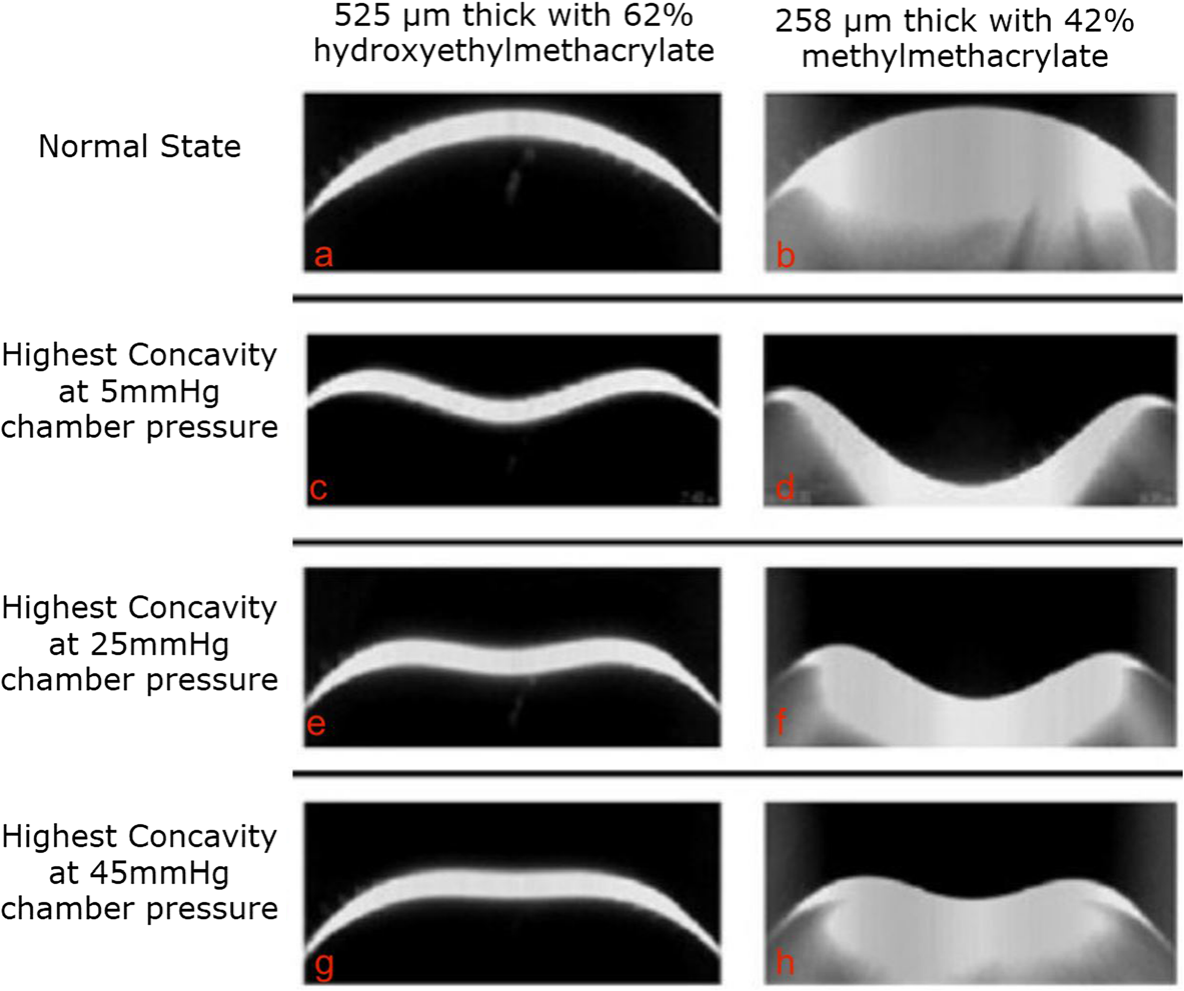
The impact of the chamber pressure on the deformation of two different contact lenses. The toughest lens (525 μm thick with 62% hydroxyethyl methacrylate) in its natural state (**a**) is compared to the most pliable lens (258 μm thick with 42% methyl methacrylate) in its natural state (**b**). Note that each lens deforms more at higher chamber pressures and that the toughest lens deforms less when compared to the most pliable lens under the same pressure levels of 5 mmHg (**c** and **d**), 25 mmHg (**e** and **f**), and 45 mmHg (**g** and **h**). However, note the toughest lens deforms more under low pressure (**c**) than the most pliable lens under high pressure (**h**) [55]. Personal archive

An experimental study demonstrated the influence of the chamber pressures on the biomechanical response of three different contact lenses that served as corneal models. These contact lenses had a known thickness and polymer composition. Accordingly, to the analysis of the ultra-high-speed Scheimpflug imaging, material composition influences the deformation more than the thickness. Moreover, the chamber pressure had a significant impact on the deformation response of each lens (Fig. 2) [56].

The impact of IOP on corneal biomechanical performance was highlighted by Ramos and collaborators in a movie that reviewed the relevance of this technology in different clinical applications (Scheimpflug Revelations). Mazzeo and collaborators reported a case of bilateral post-LASIK ectasia associated with pigmentary glaucoma in which the IOP was underestimated by Goldmann’s applanation tonometry (18 mmHg in both eyes). The ORA detected ocular hypertension with IOPcc (ORA) being 47.8 mmHg OD and 43.8 mmHg OS. With the Corvis ST, the biomechanically-corrected IOP (bIOP), developed to reduce the effect of stiffness on IOP estimates, was 62.9 mmHg OD and higher than 70 mmHg OS [57, 58]. Also, Faria-Correia and coworkers reported a case of pressure-induced stromal keratopathy that stressed the relevance of biomechanically-corrected IOP measurements for identifying ocular hypertension and noted that the IOP measurement with Goldmann tonometer was substantially smaller than the Corvis ST [55]. In both cases, the influence of IOP on the corneal deformation response was notable, considering the change in the DCR parameters after reducing IOP [55, 57].

**Fig. 3:**
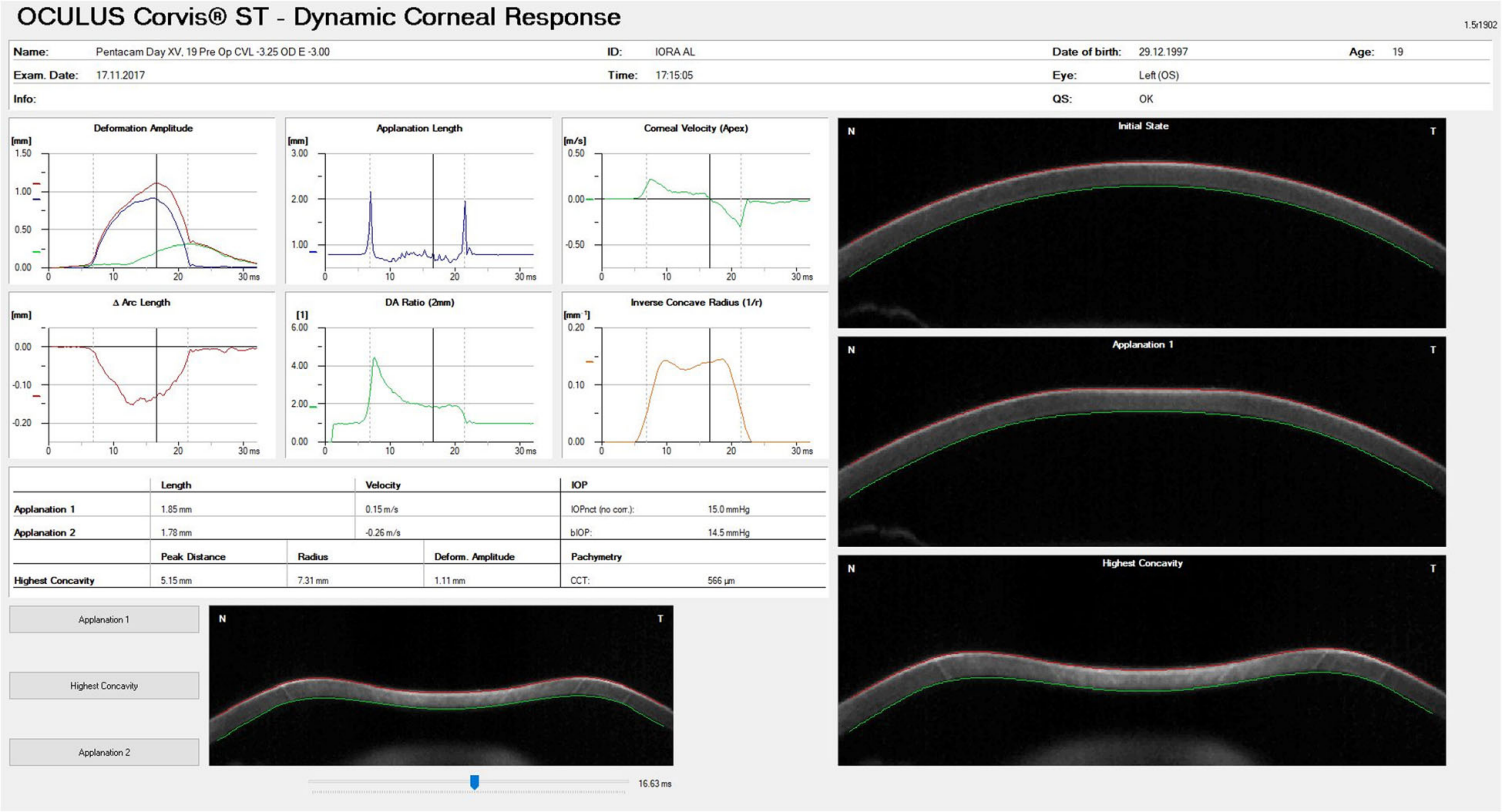
Standard Corvis ST parameters. The figure shows the deformation amplitude (DA), applanation lengths (AL), corneal velocities (CVel) recorded during ingoing and outgoing phases and the radius of curvature at the highest concavity (Curvature radius HC), and thereby calculating and registering corneal thickness and IOP. Personal archive

**Fig. 4:**
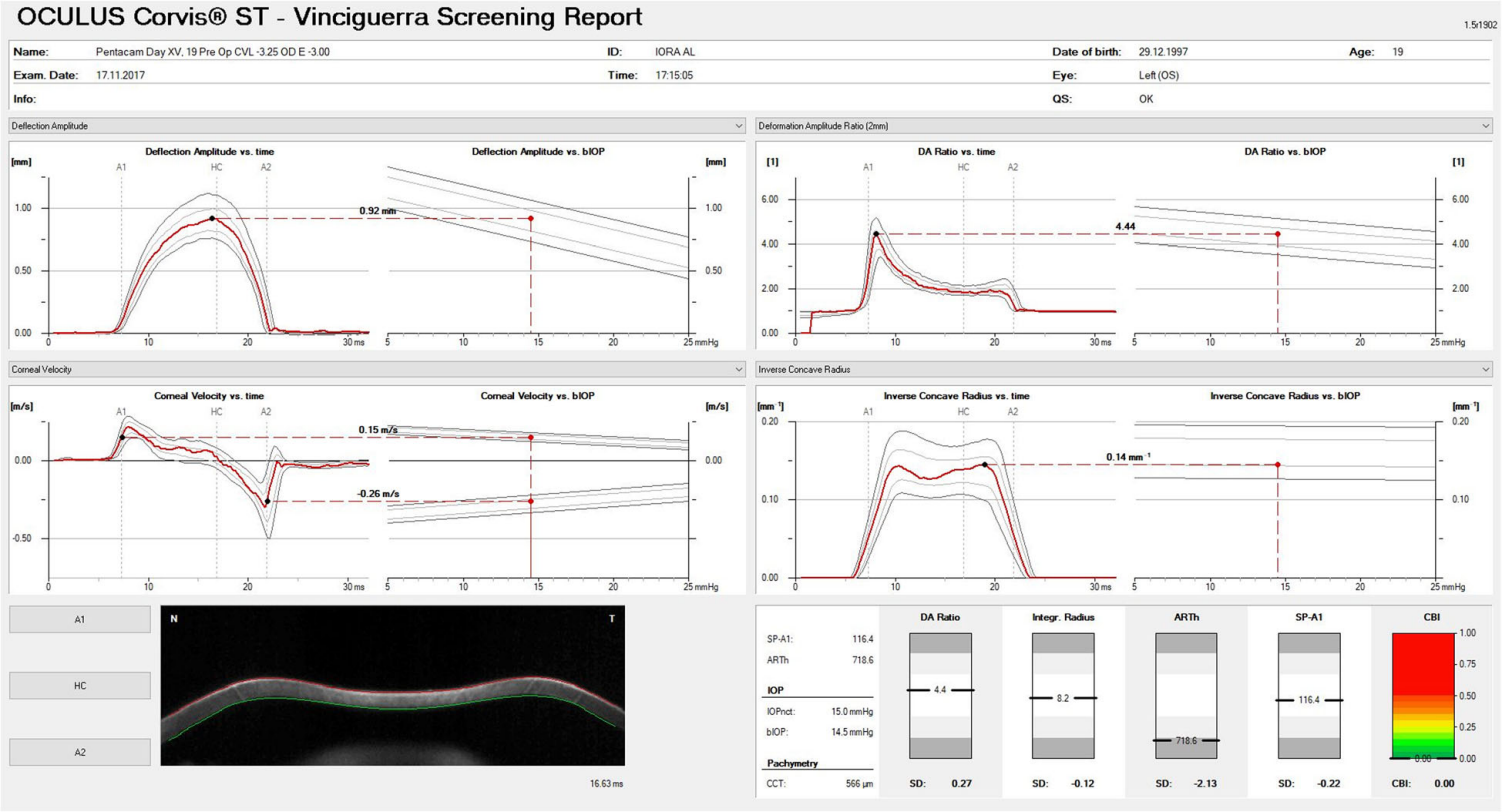
The Vinciguerra Screening Report. This display provides correlations of normality values and a biomechanically adjusted intraocular pressure. It uses a calibration factor to calculate the IOP value based on the pressure at the time of the first applanation. It empowers the calculation of the Ambrósio Relational Thickness over the horizontal meridian (ARTh) and the Corvis Biomechanical Index (CBI). Personal archive

The first generation measurement parameters of the Corvis ST provided a performance similar to that obtained by the pressure-derived ORA data for discriminating healthy and KC eyes [59, 60]. However, the more substantial details of the DCR by the Scheimpflug camera enabled the development of new parameters that consider the IOP influence on the DCR parameters (Fig. 3). These metrics have demonstrated a superior ability to detect the onset of ectatic disease [61–64]. In 2014, a multicentric international investigation group was created. The goal was to improve knowledge about Corvis ST technology with a distinctive focus on the investigation of the ectatic corneal disease using Scheimpflug imaging [13, 65, 66]. One of the outcomes of this collaborative work was the Vinciguerra Screening Report (Fig. 4). It provided correlations of normality values and a biomechanically-corrected IOP. Another outcome was the bIOP, which was developed through a finite element parametric study, using central cornea thickness and age in addition to deformation response parameters to reduce the effect of stiffness on IOP estimates [58, 67]. The bIOP correction has been successful in providing close estimates of true IOP in ex vivo tests conducted on human donor eye globes and in reducing association with the cornea’s thickness and age [68].

The horizontal Scheimpflug image of the undisturbed cornea also provides data for calculating the profile or the proportion of increase of corneal thickness from the apex towards the nasal and temporal sides. The characterization of the thickness data on the horizontal Scheimpflug image (the division between corneal thickness at the thinnest point and the Pachymetric Progression Index) enables the calculation of the Ambrósio Relational Thickness over the horizontal meridian (ARTh) [69]. The investigators used linear regression analysis to combine ARTh with corneal deformation parameters to generate the Corvis Biomechanical Index (CBI) [70]. Vinciguerra and coworkers demonstrated that a cut off value of 0.5 CBI was able to correctly identify 98.2% of keratoconic cases among normal with 100% specificity [70].

**Fig. 5:**
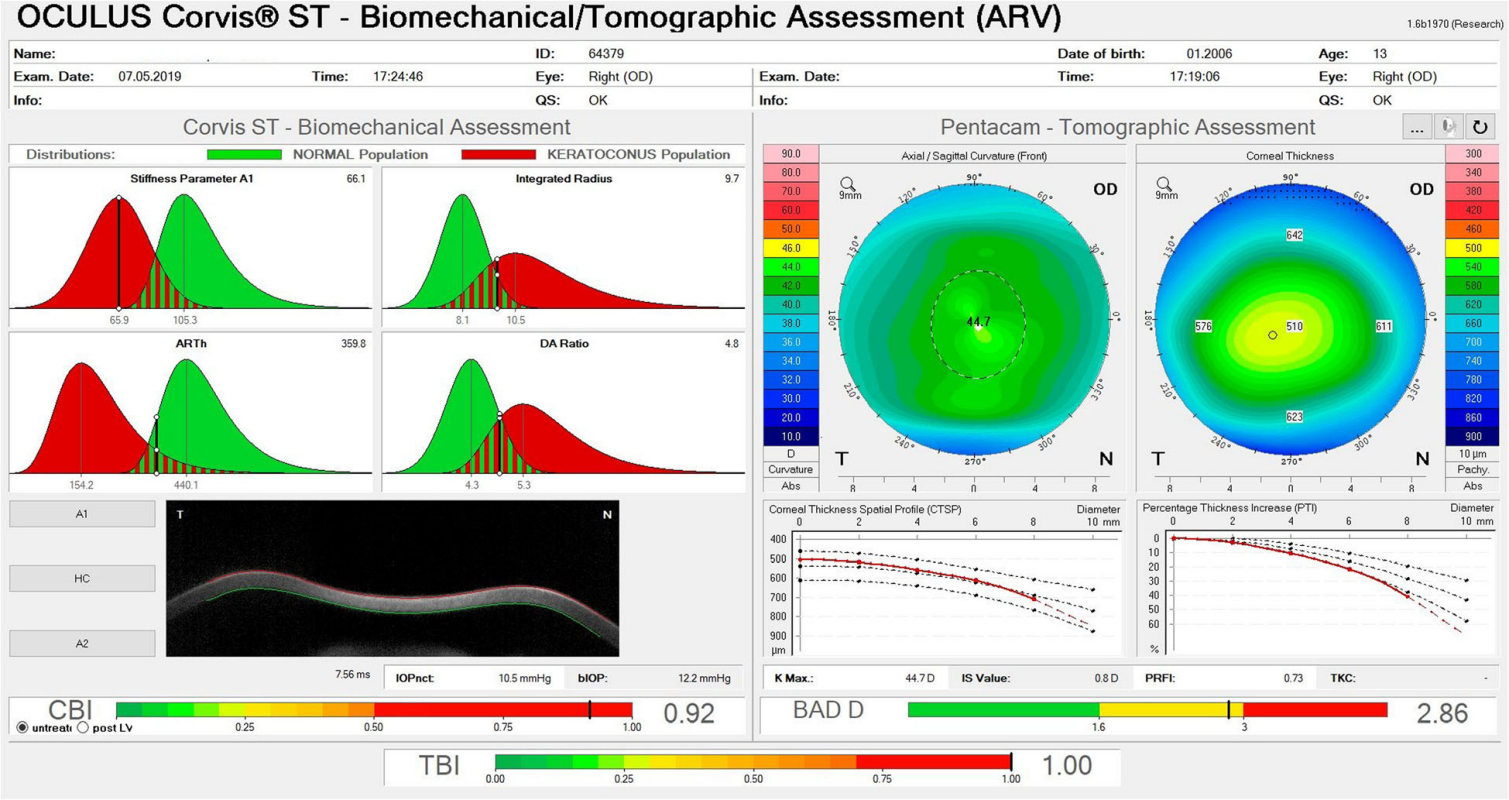
The ARV (Ambrósio, Roberts & Vinciguerra) Biomechanical and Tomographic Display showing the Corvis Biomechanical Index (CBI), tomographic biomechanical index (TBI) from the VAE-NT case with uncorrected distance visual acuity of 20/20. Personal archive

**Fig. 6:**
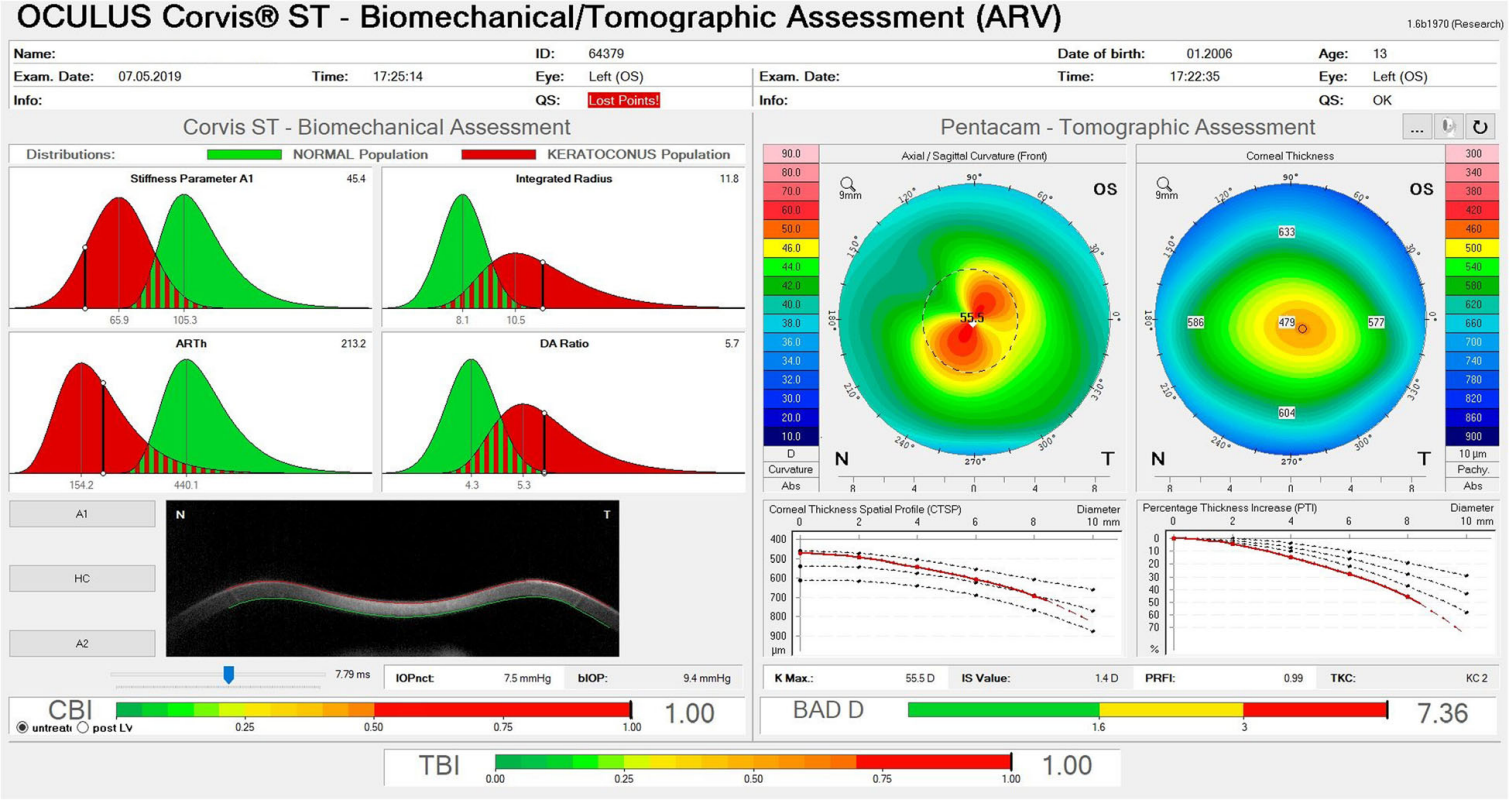
The Ambrósio, Roberts & Vinciguerra (ARV) Display from the VAE-E (fellow eye of the eye on Fig. 5). Personal archive

Subsequently, Ambrósio and coworkers continued this multicenter study to enhance ectasia detection and used artificial intelligence to develop a new index combining tomographic and biomechanical data, the tomographic biomechanical index (TBI) [13, 17]. This study involved one eye randomly selected from each of the 480 normal patients, 204 “bilateral” KC cases, and 72 unoperated ectatic eyes (VAE-E) from 94 (VAE-NT) patients with very asymmetric ectasia, who presented fellow eyes with normal topographic maps based on rigorous objective criteria. The random forest will leave-one-out cross validation using the best machine learning function for the TBI. The cutoff of 0.79 provided 100% sensitivity and specificity to detect clinical ectasia (KC + VAE-E cases). For the eyes with a normal topographic pattern, an optimized cutoff of 0.29 provided 90.4% sensitivity and 96% specificity with an area under the ROC curve of 0.985 [17]. Figures 5 and 6 illustrate the combined Ambrósio, Roberts, and Vinciguerra Display from a very asymmetric ectasia patient seen after the TBI was developed.

Various external validation studies were conducted demonstrating that the TBI had the ability to detect mild forms of ectasia in VAE-NT cases (Table 3) [16, 61, 62, 71, 75]. While some of these studies have found a relatively lower sensitivity for the VAE-NT eyes (some with NTT - normal topography and tomography), it is essential to note that some of these cases may be truly unilateral ectasia due to mechanical trauma [76, 77]. An optimized artificial intelligence function is under development using a larger population dataset for training.

**Table 3:** Tomographic biomechanical index (TBI) clinical studies

Author / Reference	NE (n)	Clin Ectasia (n)	Cut-off	Sensitivity (%)	Specificity (%)	AUC	VAE-NT	Cut-off	Sensitivity (%)	Specificity (%)	AUC	Observation
Steinberg J et al. [71]	105	96	–	98.00	100	0.998	32	0.11	72.00	71.00	0.825	VAE NTT: 18 eyes Sensitivity: 67% / Specificity: 65% / AUC: 0.732
Kataria P et al. [62]	100	100	> 0.63	99.00	100	0.995	100	> 0.09	82.00	78.00	0.793	–
Ferreira-Mendes J et al. [16]	312	118	0.335	94.40	94.90	0.988	57	0.295	89.50	91.00	0.96	–
Chan TCY et al. [72]	37	23	–	–	–	–	–	0.16	84.40	82.40	0.925	–
Sedaghat MR et al. [61]	137	145	> 0.49	100	100	1.000	–	–	–	–	–	–
Koc M et al. [73]	35	–	–	–	–	–	21	0.29	67.00	86.00	0.790	–
Koh S et al. [74]	70	–	–	–	–	–	23	> 0.259	52.17	88.57	0.751	–

*NE=* normal eyes, *VAE-NT=* very asymmetric eyes with normal topography, *NTT=* eyes with normal topography and tomography, *AUC=* area under the receiver operating characteristics curve

The TBI has been proposed to epitomize the intrinsic ectasia susceptibility for ectasia progression. Shetty and coworkers reported a case of ectasia after small incision lenticule extraction (SMILE) that was classified preoperatively as normal considering a standard evaluation [78]. Remarkably, the retrospectively calculated TBI was within the range of abnormality, indicating moderate ectasia susceptibility [79]. Besides the TBI data, the SMILE lenticules from both eyes of this patient that developed ectasia were retrieved and compared with five eyes from three stable-SMILE patients that were matched for age, sex, and duration of follow-up. Gene expression analysis demonstrated reduced expression of lysyl oxidase (LOX) and collagen types I alpha 1 (COLIA1) in the SMILE lenticules that developed ectasia, which may point to the confirmation of clinical predisposition for ectasia development in the molecular domain, confirming ectasia susceptibility [78].

The Corvis presents a parameter that serves as a biomarker for corneal stiffness, called the SP-A1. It is the result of dividing the loading (air pressure minus bIOP) on the cornea by the displacement of the corneal apex at the first applanation moment. The SP-A1 value was reported to be lower in thinner than in normal corneas [70]. Interestingly, SP-A1 has negative correlation with the corneal back-scattering (referred to as densitometry) values. This implies that, among patients with KC, increased corneal densitometry values may indicate compromised corneal stiffness [80, 81].

Multiple parameters were combined (A1 velocity, DA, DA Ratio Max 1 mm, Max Inverse Radius, and SP-A1) to evaluate and compare corneal biomechanical response and it was concluded that into a logistic regression equation it allows for high sensitivity and specificity for distinguishing normal and keratoconic eyes [82]. A study of the two-year changes in corneal stiffness parameters (SP-A1) after accelerated collagen cross-linking (CXL) using Corvis-ST provided biomechanical evidence “in vivo” of the change in corneal response that may occur following CXL treatment [83].

**Fig. 7:**
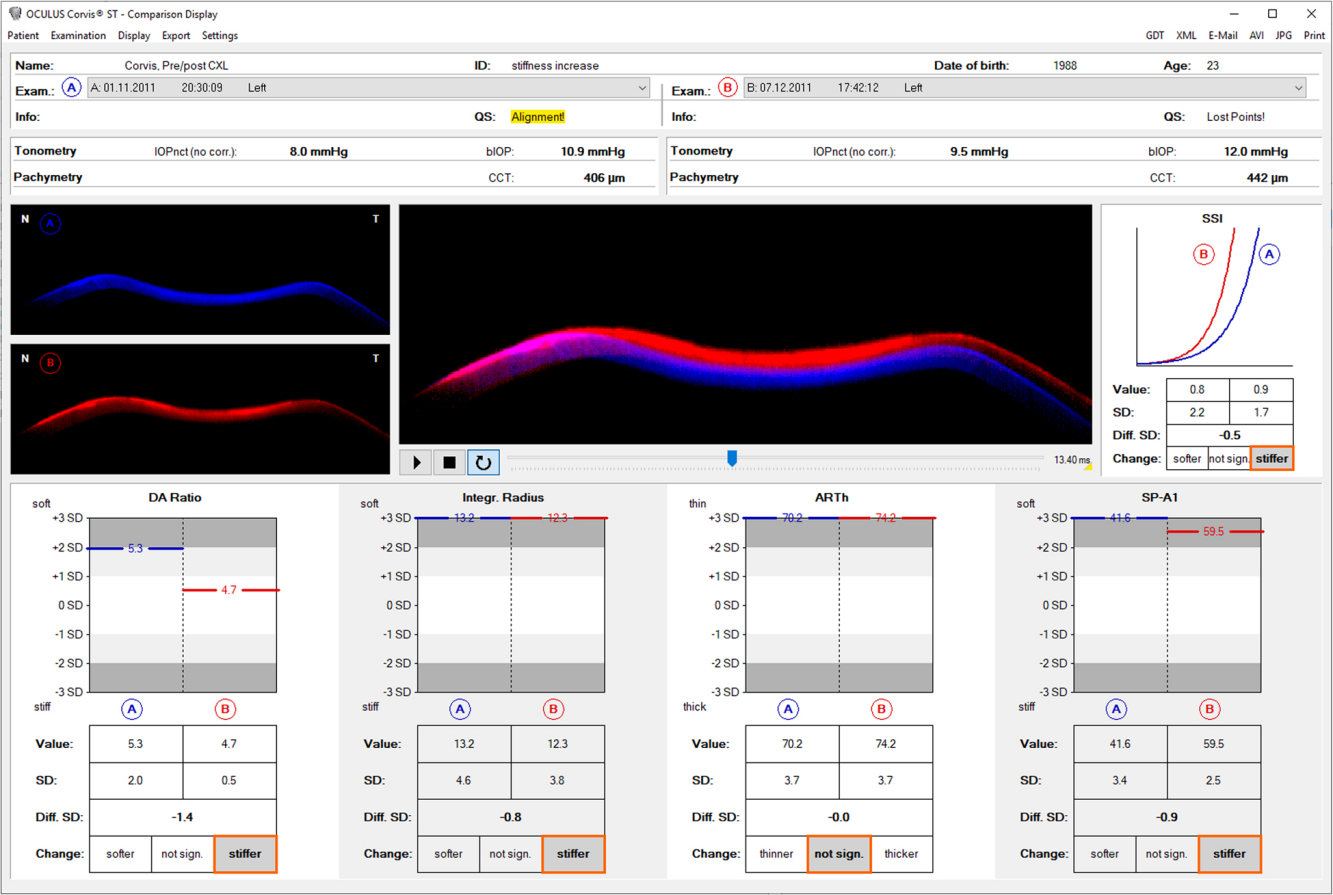
Comparative Corvis ST display before (A in red) and after CXL (B in blue), including the overlap image at higher deformation, the SSI (Stress-Stain Index), and the stress-strain curves, along with comparative DA ratio, integrated radius, and the Stiffness Parameter at first Applanation (SPA1) indicating stiffer behavior after the procedure. Personal archive

A more recent development was the introduction of the SSI (Stress-Stain Index) algorithm, which was generated based on predictions of corneal behavior using finite element models simulating the effects of IOP and the Corvis ST air puff. It was the first standard mechanic metric that could be derived in vivo, allowing to build the whole stress-strain curve of corneal tissue. Besides the detection of patients with higher risk or susceptibility for ectasia development or progression after refractive surgery, the SSI may provide clinical documentation for the biomechanical changes after cross-linking procedures (Fig. 7) [67].

There are parameters measured by the Corvis ST that are viable to discriminate healthy from keratoconic corneas, and also crosslinked from non-crosslinked keratoconic corneas. These parameters include the applanation velocity 2 (A2V), that is the velocity of corneal apex during the second applanation, and the second applanation length (A2L), which measures the cord length of A2. The difference between the first applanation length (A1L), that is the cord length of A1, and A2L could consistently discriminate crosslinked from non-crosslinked and healthy corneas, which illustrates the potential of the Corvis ST in monitoring corneal changes after crosslinking treatment [84].

Other approaches that combine corneal deformation analysis with high-speed imaging have been proposed, such as swept-source OCT or supersonic shear-wave imaging technology [5, 41, 85]. OCT topography of the Bowman’s layer significantly improved the detection of forme fruste KC with artificial intelligence [86].

### Supersonic shear-wave imaging

Tanter and collaborators evaluated the ability of ultrafast and high-resolution ultrasonic systems to provide a real-time and quantitative mapping of corneal viscoelasticity in ex vivo porcine cornea using the supersonic shear imaging technique. The technique includes a dedicated ultrasonic sequence that combines the generation of remote palpation in the cornea and ultrafast (20,000 frames/s) ultrasonic imaging of the resulting corneal displacements that evolve into a shear wave propagation whose local speed was directly linked to local elasticity. The authors concluded that supersonic shear imaging technique could construct in real-time noninvasive, high-resolution, and quantitative maps of whole corneal elasticity [87].

### Surface wave elastometry

This method is a nondestructive technique for characterization of corneal stiffness with measurement precision [88]. Dupps and collaborators used a handheld prototype system to measure ultrasound surface wave propagation time between two fixed-distance transducers along with a ten-position map in porcine corneas and human donor eyes. They concluded that this technique in in vitro experiments allows focal assessment of corneal biomechanical properties that are relevant in refractive surgery, ectatic disease, and glaucoma [88].

### Elastography with gonioscopy lens

This method consists of a scanner that provides a highly regular scan profile over a range sufficient to image the entire width of the cornea and a portion of the sclera in a single scan [89]. Ford and collaborators presented 2-D pan-corneal deformation maps in human donor eye that were acquired with no exogenous tissue contrast and with a stressor akin to clinical applanation tonometry or gonioscopy that can be performed without significant increases in IOP. The displacement behavior was resolvable in time, which allowed for the determination of viscoelastic behavior [89].

This technique is nondestructive and provides spatial property information at physiological levels of stress without separating ocular tissue from its natural mechanical boundary conditions, so it has excellent potential for implementation in vivo, and is capable of resolving minimal displacement differences in corneal tissue that may provide significant sensitivity advantages for early detection of ectatic disease [89].

### Brillouin optical microscopy

Brillouin optical microscopy was recently introduced to measure corneal biomechanics in vivo through the analysis of light scatter and mapping the biomechanical state of the cornea with 3-D capability. The method can determine intrinsic viscoelastic properties decoupled from structural information and applied pressure [90, 91].

The cornea has a nonlinear stress-strain behavior, which confirms that the tissue does not have a constant modulus. The tangent modulus increases gradually with stress or applied pressure [92]. Seiler and coworkers demonstrated the impact of age on corneal stiffness findings by Brillouin spectroscopy and found statistically significant differences when comparing normal and keratoconic corneas. However, the accuracy of the first reported findings is relatively weak [93].

## Conclusions

Corneal biomechanics is a subject of tremendous interest for clinical research in modern ophthalmology. There are novel tools, such as the Brillouin optical microscopy, which provide information about corneal biomechanical properties. However, most of the clinical data is related to the biomechanical response to non-contact tonometry. Despite the substantial developments over the last two decades, in vivo characterization of corneal biomechanical response is influenced by IOP. However, novel developments, such as the Stress-Strain Index, provided by the Corvis ST was successful in estimating stiffening following CXL treatment [67].

Knowledge of corneal biomechanics would be useful in several clinical applications, including management of glaucoma, ectasia risk-profiling, and the degree and depth of CXL [6–8]. The integration of tomographic and biomechanical data has demonstrated potential to improve the accuracy of detection of ectatic disease and identify susceptibility to develop this complication after laser vision correction [11, 15–17]. Further integration with other data, such as ocular wavefront, axial length, segmental layered (epithelium) and microlayer (Bowman) tomography is also promising. We do foresee continuous and accelerated research and development in this field that will further integrate multimodal corneal imaging, biomechanics, molecular biology, and genetics. In this environment with an overwhelming amount of clinical data, artificial intelligence will play a fundamental role so that we can augment the efficacy of patient care.

## Data Availability

Not applicable.
